# MCP: Multi-Chicken Pose Estimation Based on Transfer Learning

**DOI:** 10.3390/ani14121774

**Published:** 2024-06-12

**Authors:** Cheng Fang, Zhenlong Wu, Haikun Zheng, Jikang Yang, Chuang Ma, Tiemin Zhang

**Affiliations:** 1 College of Engineering, South China Agricultural University, 483 Wushan Road, Guangzhou 510642, China; gu5457111@gmail.com (C.F.);; 2 National Engineering Research Center for Breeding Swine Industry, Guangzhou 510642, China; 3 Guangdong Laboratory for Lingnan Modern Agriculture, Guangzhou 510642, China

**Keywords:** chicken, multi-objective, top-down, pose estimation, transfer learning

## Abstract

**Simple Summary:**

This study introduces a novel system called MCP for estimating the poses of multiple chickens using advanced deep learning techniques. Through automatically detecting and analyzing the postures of chickens, the MCP system helps to assess their behavior and welfare more effectively. The developed system uses a method known as transfer learning, which improves its accuracy and efficiency. The test results show that our system performs well, making it a valuable tool for poultry managers and researchers aiming to enhance animal welfare through technology. This work paves the way for future advancements in animal behavior analysis and could be adapted to other species as well.

**Abstract:**

Poultry managers can better understand the state of poultry through poultry behavior analysis. As one of the key steps in behavior analysis, the accurate estimation of poultry posture is the focus of this research. This study mainly analyzes a top-down pose estimation method of multiple chickens. Therefore, we propose the “multi-chicken pose” (MCP), a pose estimation system for multiple chickens through deep learning. Firstly, we find the position of each chicken from the image via the chicken detector; then, an estimate of the pose of each chicken is made using a pose estimation network, which is based on transfer learning. On this basis, the pixel error (PE), root mean square error (RMSE), and image quantity distribution of key points are analyzed according to the improved chicken keypoint similarity (CKS). The experimental results show that the algorithm scores in different evaluation metrics are a mean average precision (mAP) of 0.652, a mean average recall (mAR) of 0.742, a percentage of correct keypoints (PCKs) of 0.789, and an RMSE of 17.30 pixels. To the best of our knowledge, this is the first time that transfer learning has been used for the pose estimation of multiple chickens as objects. The method can provide a new path for future poultry behavior analysis

## 1. Introduction

Animal health and welfare is a key concern for animal researchers [1]. Animal postures and their corresponding behaviors contain important information that allows managers to better assess the health and welfare of animals [2]. One of the most common methods for monitoring animal posture behavior is the use of sensor technology [3]; however, in animal posture research, contact sensors may cause a stress response. Computer vision has the advantage of no contact, which can reduce the influence on animal posture behavior [4,5]. Studying animal posture often requires analyzing offline videos, but manually analyzing these offline videos is a time- and labor-intensive task [6]. Therefore, automated pose estimation methods and analysis tools can improve the efficiency of animal researchers [7,8].

Before deep learning flourished, traditional animal pose estimation was measured by placing markers on the target animal [9], using a body model with edge features [10], or using decoders with artificial features [11].

In recent years, the rapid popularity of human pose estimation methods based on deep learning has made animal pose estimation possible. Human pose estimation is a computer vision technology that detects and recognizes human body joint positions and their relationships using algorithms to reconstruct human postures [12,13,14]. It is widely used in action recognition [15], virtual reality [16], behavior analysis [17], and sports monitoring [18]. It typically involves detecting joints and constructing the human skeleton using 2D images or 3D data from cameras. Deep learning methods, such as convolutional neural networks (CNNs) and graph convolutional networks (GCNs), have greatly improved the accuracy and robustness of pose estimation, enabling real-time and high-precision applications [19,20]. Therefore, more and more animal researchers are trying to use deep learning methods to estimate animal poses, and much great progress has been made.

Mathis et al. successfully applied a convolutional neural network (CNN) to human and animal pose estimation and developed deeplabcut, which can analyze the frame of an animal pose [21]. Pereira et al. developed the LEAP pose estimation software (Ver. 1.0) to analyze animal poses, and verified the performance of the software using fruit fly images [22]. Graving et al. developed a software platform called DeepPoseKit (Ver. 1.0) based on a GPU fast peak detection algorithm to estimate animal postures automatically [23]. Li et al. compared various pose estimation networks and found that a stacked hourglass network can obtain good results in the estimation of cattle body postures [24]. Zhu et al. used the improved two-stream RGB-D Faster R-CNN algorithm to automatically identify five postures of lactating sows, among which the average precision of standing, sitting, sternal recumbency, ventral recumbency, and lateral decubitus recumbency were 99.74, 96.49, 90.77, 90.91, and 99.45% [25]. Marshall et al. developed the CAPTURE platform to automatically track mouse posture behavior [26].

For multi-animal pose estimation, Pereira et al. proposed SLEAP, a multi-animal pose estimation system that can track and estimate the poses of two animals (1024 × 1024) at a speed of 320 frames per second (FPS) [27]. Chen et al. developed a tracking system called AlphaTracker to study social interaction between mice [28]. Walter et al. proposed a rapid multi-animal pose estimation system called TRex, which can track and identify more than 100 unmarked individuals [29]. Lauer et al. used the improved DeepLabCut algorithm to analyze the parenting behavior of adult mice, marmoset home-cage behavior, and fish schooling behavior [30].

Another study focused on extracting a chicken’s posture and the geometric configuration of multiple body parts [31]. The initial work on animal pose estimation was all about extending deep learning algorithms designed for human pose estimation. However, there are few published studies on the multi-objective pose estimation of poultry, so we aimed to estimate the poses of poultry using a deep learning-based pose estimation algorithm. In this study, we re-study multi-person pose estimation and apply it to multi-chicken pose estimation. The model is mainly composed of two steps: the first step is a multi-chicken detection module, and the second step is a single-chicken pose estimation module. Combining these two modules, the model can estimate the pose of multiple chickens.

The main contributions of this study are as follows:

The study proposes a multi-chicken pose estimation system based on transfer learning called MCP, which adopts a top-down mode to automatically estimate the pose of each chicken in the image.

The pose estimation algorithm proposes a CKS index to evaluate the similarity degree of chicken keypoints through improving the object keypoint similarity (OKS) index.

A lower RMSE of the pixels is beneficial to the subsequent analysis of a chicken’s motion and behavior.

## 2. Materials and Methods

### 2.1. Experimental Environment

The experiment in this study was conducted at a poultry farm in Gaoming District, Foshan City, Guangdong Province, China. In the study, a high-definition camera (Logitech C922, Europe SA, Lausanne, Switzerland) was used to capture video images of chickens. The experiment object was K90 jute broiler chickens, which were between 40 and 70 weeks old. The captured video frame rate was 30 FPS, and the resolution was 1920 × 1080 pixels. As a low-cost product, this camera can be used in practical livestock environments. A total of 29 videos, including multiple chickens, were collected in this experiment. The experiment was performed in accordance with the guidelines approved by the Experimental Animal Administration and Ethics Committee of South China Agriculture University (SYXK-2019-0136).

### 2.2. Data Preprocessing

**Figure 1:**
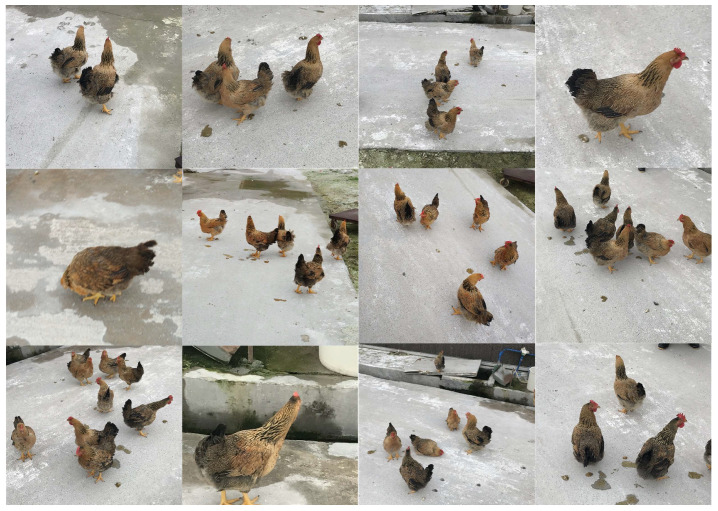
A portion of the images in the data set.

The chicken data set was constructed from 29 videos, including 3831 images in total. Figure 1 shows some images from the data set. The images included instances of single and multiple chickens, and the data set was randomly mixed into a training set, a validation set, and a test set. In order to reduce the memory consumption of GPU during training, all images collected were preprocessed using OpenCV and adjusted to a resolution of 640 × 640 pixels. Among them, the ratio of the training set, verification set, and test set was 8:1:1, respectively (3063/384/384), used for network training/verification/testing. In order to achieve accurate multi-chicken pose estimation results, we needed to annotate the images for network learning. In the selection of chicken keypoints, the reference pose selected was composed of 10 keypoints [32]. In all pictures, the ground truth (GT), keypoints, and the connection between the keypoints of each chicken were marked for data modeling.

### 2.3. Top-Down and Bottom-Up Mode

Traditional multi-person pose estimation is classified according to abstract features and pixel features, which can be divided into top-down and bottom-up modes. Similarly, the multi-chicken pose estimation process can also be divided into top-down and bottom-up modes according to the priority of the abstract features and pixel features.

**Figure 2:**
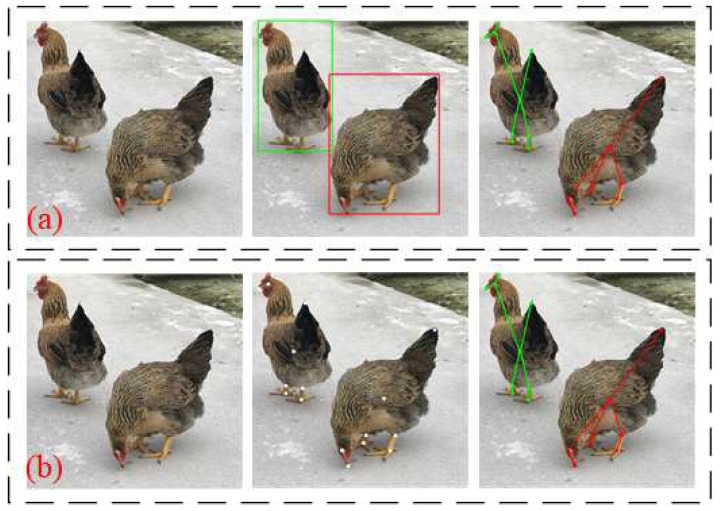
Top-down and bottom-up modes. (**a**) Top-down mode; (**b**) bottom-up mode.

As shown in Figure 2, the top-down mode starts with high-level abstract features, first detecting the chicken and generating the chicken’s position within the bounding box (Bbox). Then, the pose estimation of the chicken in each Bbox was made. In contrast, the bottom-up mode starts with low-level pixel features, first predicting the locations of all the joints or keypoints for each bird in the input image and then grouping these key points through chicken model fitting or other algorithms to determine which bird these keypoints belong to.

### 2.4. The Basic Structure of MCP Algorithms

**Figure 3:**
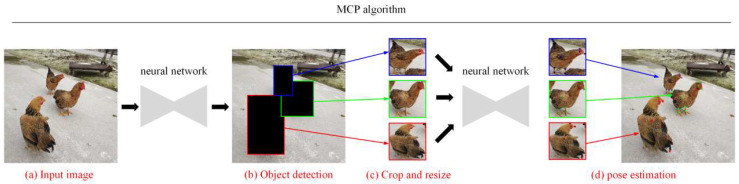
MCP pose estimation system. (**a**) Input image; (**b**) object detection; (**c**) crop and resize; (**d**) pose estimation.

All of the algorithms used in this study were written in the Python language. As shown in Figure 3, the MCP pose estimation system is mainly divided into two steps. The first step is a multi-chicken detection module, and the second step is a single-chicken pose estimation module.

Firstly, a Bbox was generated around each chicken using the multi-chicken detector. Then, the Bboxes were cropped and adjusted to the same size. Finally, these Bboxes were input into the pose estimation network, and, finally, the pose of each chicken was obtained.

#### 2.4.1. The Basic Structure of the MCP Algorithms

**Figure 4:**
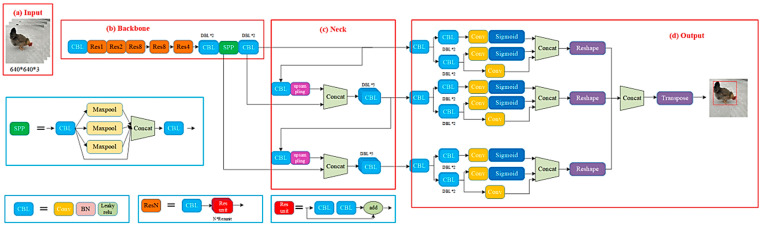
YOLOX network. (**a**) Input; (**b**) backbone; (**c**) neck; (**d**) output.

In the study, the detector used for multi-chicken detection was YOLOX, which is an open-source object detector (Megvii; 2021) [33]. The YOLOX-Darknet53 backbone network neck structure is the same as YOLOV3. The YOLOX network is shown in Figure 4.

**Figure 5:**
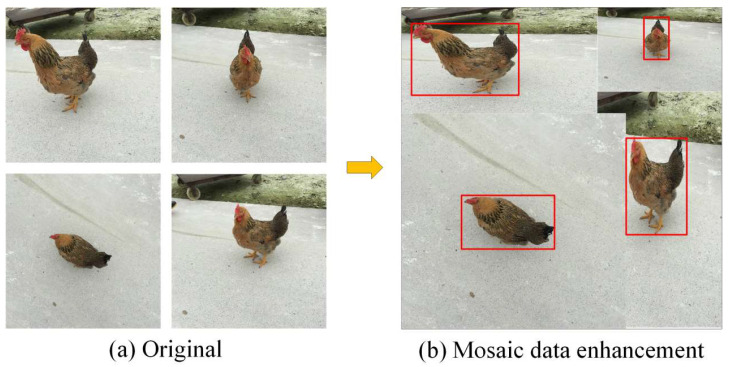
Mosaic data enhancement strategy. (**a**) Original image; (**b**) after enhancement.

On the input side of the network, YOLOX mainly adopts the Mosaic and MixUp data-enhancement methods. The Mosaic data-enhancement method mainly performs splicing via random scaling, random clipping, and random arrangements, which has a good detection and improvement effect for small objects. Figure 5 shows a typical example of Mosaic data enhancement.

**Figure 6:**
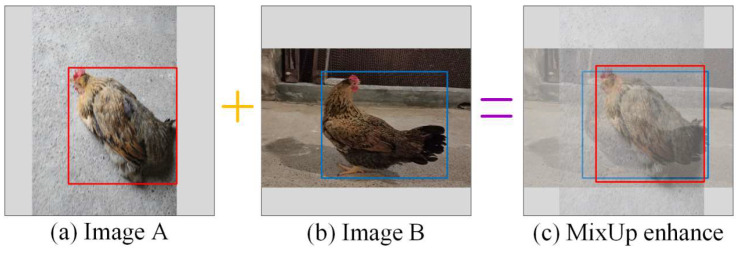
MixUp data enhancement strategy. (**a**) Image A; (**b**) image B; (**c**) after enhancement.

MixUp is an additional enhancement strategy that builds on the Mosaic process. Figure 6 shows a typical example of MixUp data enhancement.

#### 2.4.2. Single-Chicken Pose Estimation

**Figure 7:**
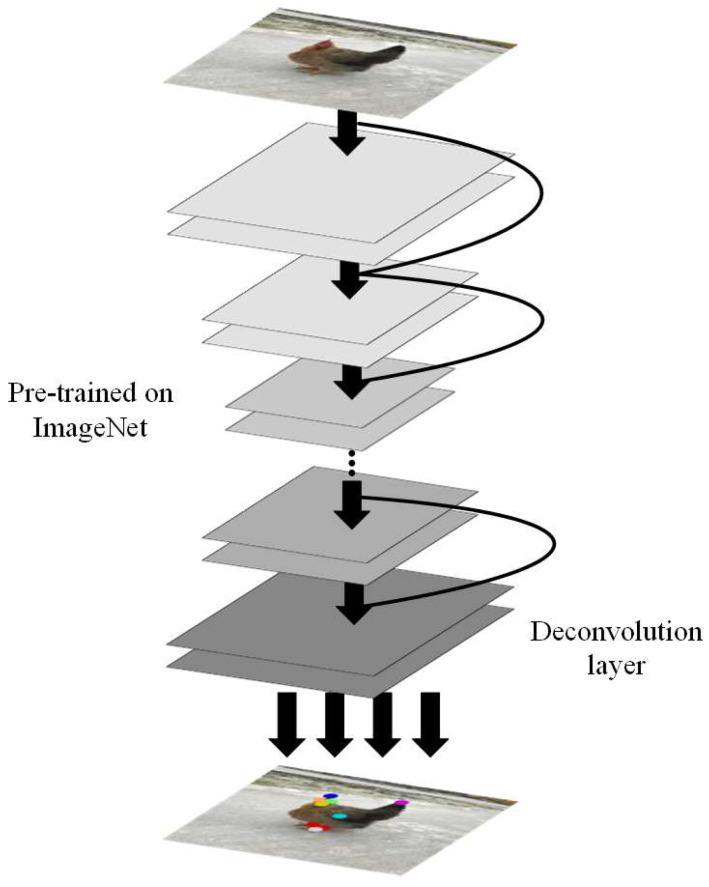
Chicken pose estimation network.

The pose estimation network is shown in Figure 7.

The pose estimation network consists of EfficientNet pretrained on ImageNet and several deconvolution layers. EfficientNet was first proposed by Google in 2019 to optimize the network for efficiency and accuracy using a composite model scaling method to balance the three dimensions of resolution, depth, and width [34]. In this study, the feature information of the chicken keypoints was extracted through EfficientNet; then, the spatial feature information of the chicken keypoints was obtained by using a deconvolution layer, and finally, the pose of the chicken was obtained by using keypoint connection. The problem with EfficientNet’s proposed composite model scaling method is as follows: (α,β,γ) is a set of parameters that require solutions, as shown in Equation (1):

(1) $$\begin{array}{c} \;\;\;\;\;\;depth:d=\alpha^{\phi }\\ \;\;\;\;\;\;width:\omega =\beta^{\phi }\\ resolution:\gamma^{\phi }\\ \;\;\;\;\;\;\;\;\;\;\;\;\;s.t.:\alpha \cdot \beta^{2}\cdot \gamma^{2}\approx 2\\ \;\;\;\;\;\;\;\;\;\;\;\;\;\alpha \geq 1,\beta \geq 1,\gamma \geq 1 \end{array}$$

where (α,β,γ) measures the proportion of the depth, width, and resolution of the network. Among them, β and γ are squared in the constraint as, if the width or resolution is doubled, the amount of calculation will increase by four times; however, if the depth is doubled, the amount of calculation will only be doubled.

### 2.5. Evaluation Metrics

In order to evaluate the accuracy of the system, CKS, *mAP*, *mAR*, and PCK were used to calculate the pose estimation effect of multiple chickens [35]. OKS was derived from the intersection over union (IoU) in object detection, which aims to calculate the similarity between human keypoints and predicted points [36]. The CKS was improved by OKS, and CKS can be expressed as per Equation (2):

(2) $${\mathrm{CKS}(B}^{T}{,B}^{P})=\sum_{i=1}^{N}e^{-\frac{{\left\|B_{i}^{T}-B_{i}^{P}\right\|}_{2}^{2}}{2\alpha \beta_{i}^{2}}}v_{i}/\sum_{i=1}^{N}v_{i}$$

where $B^{T}$ and $B^{P}$ are the true point and predicted point, respectively, with N nodes. In this research, N is equal to 10. $v_{i}$ denotes the visibility, and when this is equal to 0, this means that point i is invisible and 1 means that point i is visible. $-\frac{{\left\|B_{i}^{T}-B_{i}^{P}\right\|}_{2}^{2}}{2\alpha \beta_{i}^{2}}$ expresses the center of the true point position at the location of the detection, which is evaluated using a non-normalized Gaussian function. As a scale factor, α represents the area of chicken detection frame. $\beta_{i}^{2}$ is the normalization factor of point i of the chicken. The larger the value of $\beta_{i}^{2}$, the more difficult it is to mark point i. In the study, point body_center is equal to 0.107, and the others are equal to 0.025. Equation (2) describes the similarity relationship between GT and predicted value of a chicken. The range is [0, 1], where 0 is completely dissimilar, and 1 is completely similar.

*mAP* is an indicator that was originally described by Pascal VOC, which has been widely used in human pose estimation [37]. *mAP* calculates the GT of OKS at each threshold and predicts the value of the class true positive (*TP*) or false positive (*FP*), where the thresholds are as follows: {0.50, 0.55, 0.60, 0.65, 0.70, 0.75, 0.80, 0,85, 0.90, 0.95}:

(3) $$P=\frac{TP}{TP+FP}$$

(4) $$AP=\sum \max_{\tilde{B}\geq B}P(\tilde{B})/101_{\tilde{B}\in \{0,0.01,\ldots ,1\}}$$

(5) $$mAP=\sum_{i=1}^{10}AP/10$$

where *P* is precision and *AP* is the average of the best accuracy values for 101 thresholds.

*mAR* is defined as the mean average recall with different OKS thresholds.

(6) $$R=\frac{TP}{TP+FN}$$

(7) $$AR=\max R_{OKS_{i}}$$

(8) $$mAR=\sum_{i=1}^{10}AR/10$$

where *R* is recall, *FN* is a false negative, and *AR* represents the maximum recall rate under the current OKS.

PCK is another indicator commonly used in human pose estimation. It is usually reported as PCKh in human pose estimation, using the human head length as a normalized reference. This indicator calculates the proportion when the normalized distance between the detection keypoint and its corresponding GT is less than the set threshold.

## 3. Results and Discussion

### 3.1. Experimental Setting

The MCP presented in this study was trained on the Windows operating system (Windows 10 Pro). During training, the CUDA 11.2 and CUDNN 8.2 platforms were used, where the GPU was an NVIDIA GTX 1080TI (Python version 3.7.10 and torch version 1.7.0.) The training parameters of the experiment are shown in Table 1.

The object detection and pose estimation training parameters are shown in Table 1. In the training process, the stochastic gradient descent (SGD) optimizer was used to optimize the object detection network, and the adaptive moment estimation (Adam) optimizer was used to optimize the pose estimation network. The initial learning rate was 1 × 10^−3^, and this dropped by 50% every five steps.

### 3.2. Experimental Results

In this study, the performance of three YOLOX series detection algorithms (YOLOX, YOLOX-M, and YOLOX-S) and EfficientNet pose estimation were calculated. Table 2 shows the results of the pose estimation for multiple chickens.

The experimental results show that the mAP and mAR of the YOLOX + EfficientNet algorithm are 0.601 and 0.727, respectively. The mAP and mAR of the YOLOX-M + EfficientNet algorithm are 0.604 and 0.705, respectively, and the mAP and mAR of the YOLOX-S + EfficientNet algorithm are 0.652 and 0.742, respectively. Although the PCK score of YOLOX-S + EfficientNet was not as high as that of YOLOX-M + EfficientNet, the overall difference is not significant; the detection speed of YOLOX-S + EfficientNet was faster, and the size was smaller. In summary, YOLOX-S + EfficientNet was more suitable for the detection and pose estimation of multiple chickens.

**Figure 8:**
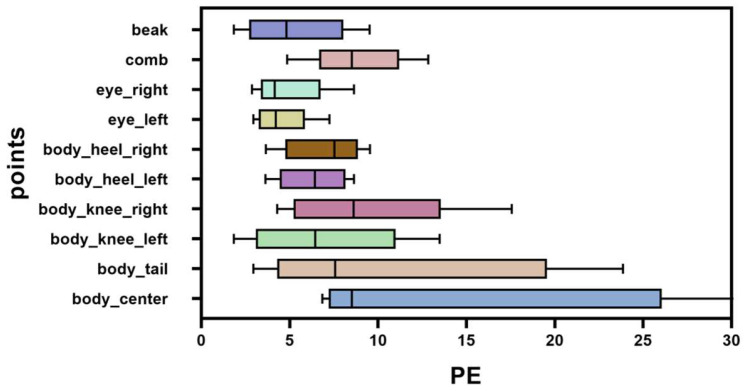
The PE of different keypoints.

Figure 8 shows the PE of different keypoints.

As shown in Figure 8, there are a total of 10 keypoints, namely body_center, body_tail, body_knee_left, body_knee_right, body_heel_left, body_heel_right, eye_left, eye_right, comb, and beak. For the chickens, we can see that some keypoints of the body were fairly accurate, while others were relatively inaccurate. In particular, due to the similarity around body_center, the keypoints near the chicken’s center are very difficult to predict, resulting in an error of 30+ pixels in the test set. Here, PE is defined as the Euclidean distance between the real point and the predicted point [38].

(9) $$PE_{i}=\sqrt{{\left(i_{realx}-i_{predictx}\right)}^{2}+{\left(i_{realy}-i_{predicty}\right)}^{2}}$$

where *i* denotes a keypoint.

**Figure 9:**
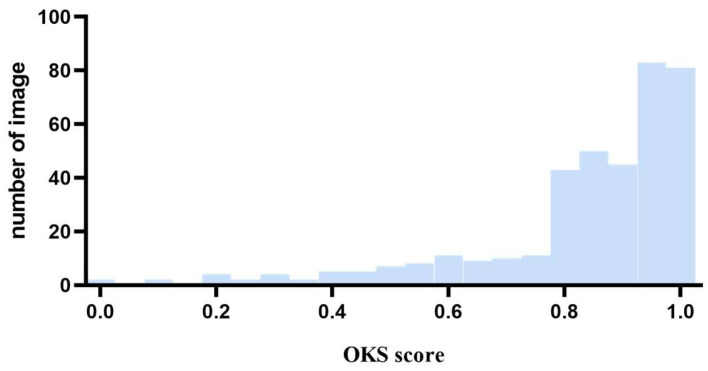
Number of images, as measured by CKS.

We conducted statistical calculations on the number of CKS score images in the test set, as shown in Figure 9.

As shown in Figure 9, although there are many images for which the CKS values are very close to 1.0, the main reason for the difference between the mAP and mAR indicators may be the number and distribution of the outliers.

In addition to the above evaluation indicators, the offset of each keypoint in the training set, verification set, and test set was also analyzed, as shown in Table 3, which is represented by pixel dispersion degree or RMSE. When compared with GT, the RMSE of the model at the predicted position is smaller, which verifies that the model proposed in this paper is reliable for detecting each keypoint.

**Figure 10:**
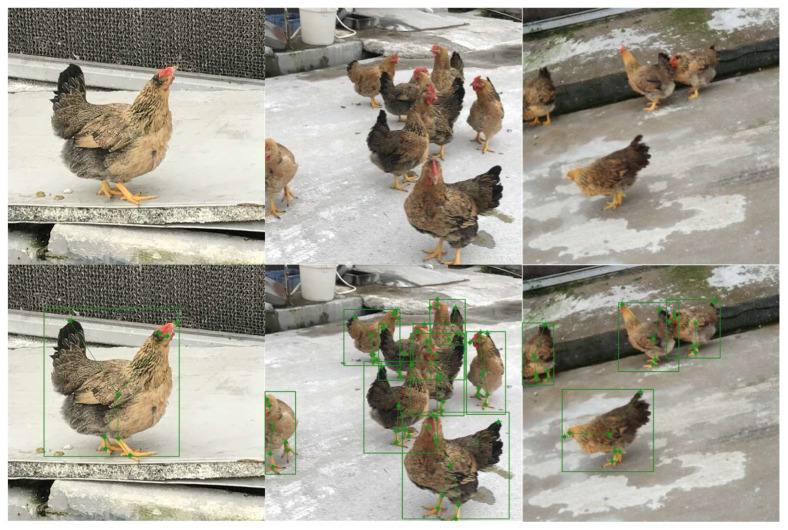
Examples of partial test results of chicken posture.

As shown in Figure 10, some results of the test set are as follows: the upper part is the original image, and the lower part is the result after MCP processing.

**Figure 11:**
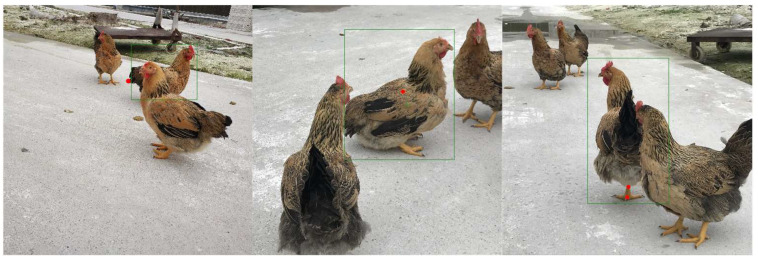
Some failure situations.

However, some chicken positions were not accurately identified or missed, as shown in Figure 11.

As shown in Figure 11, some keypoints were not fully recognised. This is because we used a top-down approach, where points near the edges may be missed due to the precision limitations of the detection algorithm. If a keypoint is not within the detection box in the first stage of detection, it can result in misdetection. Additionally, due to the similarity of points near the center, some central points were not predicted accurately. Meanwhile, if one of the chicken’s feet is occluded, the left foot may be misidentified as the right foot.

In this study, a multi-chicken pose estimation method based on transfer learning was proposed. The experimental results show that the algorithm performs well in keypoint detection. In fact, the points that are difficult to annotate during pose labeling often result in larger pixel errors during prediction. This is because the complexity and ambiguity in accurately identifying these points can lead to inconsistencies in the training data which, in turn, affects the model’s ability to predict these points with high precision [39,40]. Addressing this issue requires more advanced techniques and higher-quality annotations to reduce prediction errors and improve the overall pose estimation accuracy. The accurate pose estimation of multiple chickens can be helpful for subsequent motion and behavior analysis [41]. In the future, the accuracy of the chicken keypoints can be further improved, and the local pose of chickens can be constructed for the measurement of a chicken’s body size [42,43].

## 4. Conclusions

The study proposed MCP, a top-down pose estimation system for multiple chickens. The system can recognize the poses of multiple chickens in an image. The experimental results show that YOLOX-S + EfficientNet is suitable for multi-chicken detection and pose estimation, achieving an mAP of 0.652, an mAR of 0.742, and a PCK of 0.789, with a speed of 22.16 FPS. The RMSEs of different keypoints in the training set, validation set, and test set were 5.31, 7.56, and 17.30 pixels, respectively. Therefore, the system has certain reference significance for subsequent researchers in the field of poultry posture.

To the best of our knowledge, this is the first time that transfer learning has been used for the pose estimation of multiple chickens, but there is much more work to be carried out. For future work, such as multi-chicken pose occlusion, 3D data sets with depth information can be used to better solve the problem. In addition, the bottom-up pose estimation of multiple chickens is also a challenging task that deserves further study. Including reflections, shadowing, and low contrast as aspects of image comparison in future work will also be important for improving the robustness and accuracy of the system.

## Figures and Tables

**Table 1:** The training parameters of the experiments.

Module	Object Detection	Pose Estimation
Input size	640 × 640	512 × 512
Batch size	4	4
Epoch	300	200
Learning rate	1 × 10^−3^	1 × 10^−3^
Optimizer	SGD	Adam

**Table 2:** Performance of multi-chicken pose estimation algorithms.

Algorithm	mAP	mAR	PCK	Speed (FPS)
YOLOX + EfficientNet	0.601	0.727	0.771	20.46
YOLOX-M + EfficientNet	0.604	0.705	0.797	20.80
YOLOX-S + EfficientNet	0.652	0.742	0.789	22.16

**Table 3:** RMSE values of chicken keypoints.

Keypoint	Training Set	Verification Set	Test Set
body_center	6.84	8.52	25.76
body_tail	5.64	7.56	23.85
body_knee_left	4.32	6.44	13.48
body_knee_right	4.29	6.15	17.56
body_heel_left	3.62	6.42	7.68
body_heel_right	3.64	5.86	8.15
eye_left	2.94	3.54	4.45
eye_right	2.87	4.15	4.86
comb	4.85	8.52	12.84
beak	1.84	4.81	6.54
Average	5.31	7.56	17.30

## Data Availability

The data presented in this study are available on request from the corresponding author.
